# Examining Young Children’s Physical Activity and Sedentary Behaviors in an Exergaming Program Using Accelerometry

**DOI:** 10.3390/jcm7100302

**Published:** 2018-09-25

**Authors:** Minghui Quan, Zachary Pope, Zan Gao

**Affiliations:** 1 School of Kinesiology, Shanghai University of Sport, Shanghai 200438, China; 2 School of Public Health, University of Minnesota-Twin Cities, Minneapolis, MN 55455, USA; popex157@umn.edu; 3 School of Kinesiology, University of Minnesota-Twin Cities, Minneapolis, MN 55455, USA

**Keywords:** active video game, light physical activity, moderate-to-vigorous physical activity, sedentary behavior, sex difference

## Abstract

Exergaming has been observed to be a viable supplemental approach in promoting physical activity (PA) among children. However, whether sex differences in PA and sedentary behaviors exist during exergaming is inconsistent. Thus, this study aimed to quantify, via accelerometry, young children’s PA and sedentary behaviors during exergaming as well as examine sex differences in these PA and sedentary behaviors during gameplay. In total, 121 first- and second-grade children (mean age = 6.89 ± 0.9 years; 73 girls) were included in the analysis. Children were a part of a large 18-week parent study. Children wore ActiGraph GT1M accelerometers during exergaming play, with four measurements purposively selected from the 28 total exergaming sessions to capture children’s PA and sedentary behaviors during exergaming play. Outcome variables included mean percentages of time spent in moderate-to-vigorous PA (MVPA), light PA (LPA), and sedentary behavior during each exergaming session. One-way ANOVA was performed to determine whether there were differences in the percentage of time engaged in MVPA, LPA, and sedentary behavior during exergaming by sex. Accelerometry data indicated that children’s mean percentage of exergaming time spent in MVPA, LPA, and sedentary behavior were 19.9%, 32.9%, and 47.2%, respectively. However, no sex differences were present. Observations in this study indicated that boys and girls have similar PA levels during exergaming and suggests that features inherent to exergaming may assist in PA promotion among both sexes.

## 1. Introduction

Increased physical activity (PA) and reduced sedentary behavior among children and adolescents have been positively associated with improvements in physiological and cognitive outcomes, such as body composition, cardiorespiratory fitness, bone health, cognition, and academic achievement [1]. Consequently, it is recommended that children and adolescents accumulate at least 60 minutes (min) of moderate-to-vigorous PA (MVPA) per day [2]. However, a recent study’s observations among 6128 children aged 9–11 years from 12 countries suggested that only 44.1% of participants met the MVPA recommendation [3]. Indeed, physical inactivity, along with dietary behaviors, has been highlighted as an important determinant of overweightness and obesity among children worldwide [4]. Furthermore, evidence has suggested that physical inactivity and overweightness/obesity during childhood may track into later life [5,6], leading to higher risks of many chronic diseases including stroke, type 2 diabetes, and heart disease [7]. Thus, researchers have been investigating effective and novel methods of promoting PA among children.

Exergaming, a new generation of video games, requires players to physically interact with video games during gameplay through various arm, leg, or whole-body movements such as dancing, jogging, and kicking [8]. Given the fact that children spend a large proportion of their time engaging in screen-based sedentary behavior [9,10], exergaming’s physically active screen-based nature has been considered a promising way to promote PA levels in children and a feasible manner to facilitate population-level attenuation of the increasing obesity prevalence among children and adolescents [8,11].

Over the past decade, the beneficial health effects of exergaming have been extensively examined by researchers. Previous studies have suggested that exergaming may improve children’s PA levels and attitudes [11,12,13,14], energy expenditure [15,16], and perceived motor skills [17], and reduce sedentary screen time [18,19] and waist circumference [20] and improve body composition [21,22]. However, whether exergaming is an effective approach to promote PA levels and help children meet the PA recommendations (i.e., 60 min of MVPA per day) is still unclear. One study indicated that children had marginally higher energy expenditure during exergaming gameplay than sedentary computer games, but that such gameplay was not enough to meet the PA recommendations for children [23]. This observation was supported by subsequent reviews, which suggested that many exergames could not engage children in moderate-intensity PA [24], with most exergames only eliciting light PA [25,26]. Nevertheless, we cannot ignore exergaming’s potential role in facilitating PA and health promotion—particularly as increasing evidence has indicated that light PA (LPA) can confer health benefits [27]. Undoubtedly, it is easier for previously inactive children to accumulate more daily PA time during LPA before progressing toward greater MVPA participation. Hence, exergaming studies must also quantify children’s LPA during exergaming instead of concentrating exclusively on MVPA.

As exergaming has also been posited as a potential approach to promote PA levels among both sexes, observations of exergaming’s effectiveness at promoting PA between boys and girls are relevant, but have been mixed thus far. Some studies have reported that girls were less physically active than boys during exergaming play [23,28], while other studies did not observe a difference [29,30]. Considering the inconsistency of the observations, the relatively small sample sizes of previous studies [23,28,29,30], and the fact that girls are considered a high-priority group for PA promotion [31], more investigation of exergaming’s ability to promote PA among both sexes is needed within larger and more diverse samples.

Therefore, the current study aims to: (1) quantify young children’s PA and sedentary behaviors during exergaming using accelerometry; and (2) examine the sex differences in PA and sedentary behaviors during exergaming play. Study observations may provide a rationale for using exergaming as a supplement for more traditional PA promotion (e.g., physical education) in community-based contexts (e.g., school-based exergaming programs), particularly among girls.

## 2. Methods

### 2.1. Participants and Research Setting

A convenience sample of 158 (91 girls) first- and second-grade children, aged 6–8 years, were recruited from one Title-I Texas elementary school in the U.S. All children’s parents were fully informed of the study’s protocol, which was included on an informational sheet in children’s take-home backpacks. Parents interested in having their child participate subsequently signed an informed consent document. Furthermore, children provided assent to participate as well. No data collection occurred on any child before parental consent and child assent were obtained. Notably, all study procedures were approved by the University’s Institutional Review Board (IRB) and the school district prior to data collection commencing. Moreover, all procedures performed with the children were in accordance with the ethical standards of the institution and/or national research committee and with the 1964 Helsinki declaration and its later amendments or comparable ethical standards [32].

### 2.2. Measurements

PA levels and sedentary behaviors were assessed using ActiGraph GT1M accelerometers (ActiGraph, Pensacola, FL, USA), which have been proven to be valid and reliable in assessing PA and sedentary behaviors among children [33]. A 1-s epoch and cutoff points developed by Evenson and colleagues for children’s PA were used in the present study [34]. Sedentary behavior was corresponded to 0–100 counts per minute, with LPA and MVPA corresponding to 101–2295 counts and ≥2296 counts per minute, respectively. Outcome variables were mean percentages of time spent in MVPA, LPA, and sedentary behavior during exergaming play during each exergaming session. To elaborate, approximately 20–22 min of PA data was collected during each 30-min exergaming session, with research assistants documenting the exact gameplay start and stop times. Following data collection, PA data were cut within ActiLife software to reflect the start and stop times of exergaming gameplay, meaning the percentages of time in MVPA, LPA, and sedentary behavior reflect the 20–22-min gameplay periods. 

### 2.3. Procedures

Children in this cross-sectional study were part of a large 18-week exergaming intervention [35]. Throughout the intervention, an alternating two-week schedule was used. The first week included three days of a 30-min physical education session and two days of a 30-min exergaming session, whereas the second week was comprised of three and two days of exergaming and physical education sessions (30 min per session), respectively. Due to holidays and other unexpected class cancellations/activities, children actually participated in a total of 28 exergaming sessions and attended the exergaming sessions as a class (17–22 students per class). The accelerometers were placed on each children’s right hip at the level of the superior iliac crest using an elastic waistband during each exergaming session by trained research assistants. 

Twelve exergaming stations were set up in a large classroom, allowing up to 24 children to play the games simultaneously (i.e., each station accommodated up to two children). Each station included eight different Wii games, such as Wii Fit, Just Dance, and Wii Sports, which required children to perform a series of body movements to interact with onscreen characters and navigate through/successfully complete each game. Prior to testing, children were instructed how to play each game and were allowed to familiarize themselves with the games for 8 weeks prior to testing. During each exergaming session, a trained teacher or research assistant supervised the children during gameplay, with children transitioning between exergaming stations every 8–10 min. These transitions usually took less than two minutes, with these transitions allowing children to play all Wii games provided over the course of the school year, as the children were not allowed to pick the exergames played during these sessions. Notably, the teacher or research assistant supervising the children did not allow children to choose with whom they played exergaming, randomly assigning children to play with one another. This is important as it ensured that highly active friends did not bias exergaming PA measurements upward and, conversely, that highly sedentary friends did not bias exergaming PA measurements downward. Following this 18-week intervention, four days of accelerometer measurements were purposively selected from the 28 exergaming sessions to represent children’s regular PA and sedentary behaviors well when participating in this PA modality. It is important to note that several sessions lasted longer or shorter than originally scheduled due to school events or class activities, beyond the different time spent engaging in line-up/warm-up and cool-down activities in each session. Furthermore, it should be noted that there were two different seasons (winter and spring) during the data collection period. As such, the researchers believed it best to select two sessions from each season for each child. This ensured that any seasonality effect possibly present could be controlled for. Therefore, it was more accurate for the researchers to purposively select the representative four sessions. 

### 2.4. Data Analyses

Assuming the coefficient of variation (CV) of MVPA (CV = 0.39) based on the previous study [36], confidence level as 95%, and 8% level of precision, the required sample size was at least 90 in this study. Kolmogorov–Smirnov tests assessed normality, with variables described as the mean ± SD for normally distributed variables. Next, one-way ANOVA and Bonferroni post-hoc tests were used to determine whether sex differences existed in MVPA, LPA, and sedentary behavior time percentages. All analyses were conducted using SPSS version 22.0 (SPSS Inc.; Chicago, IL, USA), with a two-sided *p* value of ≤0.05 considered as statistically significant.

## 3. Results

Thirty-seven children were removed from the final analysis due to missing data on one or more of the purposively selected four exergaming sessions. The final sample was comprised of 37 six-year-old children (13 boys; 24 girls), 61 seven-year-old children (24 boys; 37 girls), and 23 eight-year-old children (11 boys; 12 girls). Table 1 presents descriptive statistics for children’s mean percentages of time spent in MVPA, LPA, and sedentary behavior. Accelerometer data indicated that children’s mean percentages of time spent in MVPA, LPA, and sedentary behavior were 19.86%, 32.94%, and 47.20%, respectively. Table 1 also shows the mean percentages of time that children were engaged in MVPA, LPA, and sedentary behavior during exergaming play by sex. No statistically significant sex differences were identified for MVPA, LPA, and sedentary behavior. Of note, children of any gender spent more than 50% of their time in MVPA and LPA.

## 4. Discussion

As exergaming may be a viable supplemental approach to promote children’s PA participation, it is important to objectively quantify children’s PA and sedentary behavior during exergaming gameplay. These observations indicated that regardless of sex, children spent more than 50% of exergaming session time participating in PA (MVPA and LPA).

Study observations suggest that exergaming may be able to supplement other PA promotion strategies among children (e.g., physical education, in-class activity breaks, etc.), particularly for programs with the objective to increase LPA among previously inactive children. Thus, despite the current study’s observation that the LPA percentage was greater than the MVPA percentage, reasons are present as to why exergaming may still be considered a potentially effective supplemental PA promotion strategy. First, the combined mean percentages for MVPA and LPA participation still accounted for more than 50% of each exergaming session. Although current recommendations stress that more than 50% of any PA session should be comprised of MVPA [37], the potential health benefits derived from engaging in LPA are emerging. Indeed, there is increasing evidence suggesting that LPA is independently and positively associated with improved health outcomes in children and adolescents, including reduced total body fat mass [38], some cardiometabolic risk factors [39], and improved body bone health [40], in addition to cognitive function improvements among boys [41]. Indeed, evidence has suggested the effectiveness of regular PA for health benefits—benefits partially explained by the increased energy expenditure that any intensity of PA confers versus sedentary behavior [42], but also because regular PA participation at any intensity (even LPA) results in improved cardiorespiratory health (e.g., increased arterial elasticity which lowers blood pressure, enhanced ability to deliver oxygen and other nutrients to muscles, etc.) [43]. Finally, LPA plays an important role in contributing to the children’s daily total energy expenditure (LPA vs. MVPA: around to 26.6% vs. 25.1%) [44]. Therefore, LPA promotion may still be considered an alternative strategy in improving health-related outcomes, particularly as children replace sedentary behavior with exergaming or similar technology-based programs. In fact, considering the potential effect of LPA on health-related outcomes, the updated youth PA guidelines for youth not only emphasize the importance of MVPA, but also highlight that several hours of LPA should be performed each day [2].

Exergaming is also not constrained by seasonal weather conditions, meaning this PA modality could be used to supplement daily segments such as recess or physical education when adverse weather conditions prohibit outdoor PA engagement. Finally, exergaming may act as an alternative supplement to physical education and recess as studies have indicated exergaming to increase children’s PA-related intrinsic motivation [45]—a psychosocial construct considered to be a crucial determinant in children’s long-term PA engagement [46,47]. Thus, exergaming might be employed as a “stepping stone” to promote increased PA outside of exergaming sessions, as shown in previous literature [12,48].

The current study also observed no sex differences in PA levels during exergaming gameplay. Recent studies on sex-related differences in daily PA levels noted that boys had higher PA and sedentary behaviors than girls—observations consistent across different countries such as the U.S. [49] and China [9,50]. However, exergaming gameplay did not appear to exhibit these trends. Our observations are consistent with the previous studies, indicating that there was no difference between boys and girls for total PA time [51] or average metabolic equivalent (METs) during exergaming [52]. These observations are promising as it indicates that health professionals may be able use exergaming as an appropriate PA promotion strategy for all children, regardless of sex. However, more investigations in larger samples akin to the sample in the present study are needed, as past investigations have reported sex differences for exergaming-related intrinsic motivation and enjoyment [52,53]—constructs which were significantly related to PA levels [46]. Nonetheless, the fact that exergaming developers strive to develop games which are appealing to both sexes may partially explain the current study’s observations. 

A few study limitations need to be noted for future direction. First, all participants were part of a convenience sample recruited from a single school in Texas, which limits the generalizability of the study’s observations to other populations from other geographic locations. Second, it is realized that the implementation of an exergaming program (or similar programs) is contingent upon school resources, as the setup of the exergaming classroom within the study school cost approximately US$3600. Notably, as the exergaming program was able to be implemented by one teacher coordinating up to 24 children playing on 12 different exergaming stations, this type of exergaming program does appear feasible to promote both LPA and MVPA among children. Fortunately, this configuration has been implemented in more than 750 public schools in the State of West Virginia for PA promotion [54]. Third, while this study does highlight the feasibility of implementing an exergaming program for PA promotion, other technologies may also be used in future studies (e.g., mobile devices), given the everchanging nature of youths’ current technology interests. Finally, the current study did not include an analysis of children’s anthropometric, physiological, or psychosocial outcomes—important components of overall health and wellness, which should be investigated thoroughly and in a longitudinal manner during future school-based exergaming or technology-based programs. 

## 5. Conclusions

In conclusion, the current study’s observations indicated that exergaming could facilitate LPA and MVPA in young children, with no difference in PA participation observed between sexes. Given exergaming’s cost-effectiveness and resistance to weather conditions, exergaming may be a supplemental apparatus in promoting PA among young children [55,56]. Future large studies akin to the current investigation are needed to determine exergaming effectiveness among different age groups (e.g., older children and adolescents) and settings (e.g., school-based vs. home-based) to further evaluate the effectiveness of this PA modality for promoting children’s PA.

## Figures and Tables

**Table 1:** Percentage of time spent in physical activity and sedentary behavior during exergaming.

	MVPA		LPA		Sedentary	
	Mean	SD	Mean	SD	Mean	SD
Total (*n* = 121)	19.86	7.16	32.94	6.29	47.20	10.68
Gender						
Boys (*n* = 48)	19.31	7.15	33.09	5.67	47.60	9.91
Girls (*n* = 73)	20.22	7.19	32.84	6.70	46.94	11.21

Note: LPA = light physical activity; MVPA = moderate-to-vigorous physical activity; SD = standard deviation.
